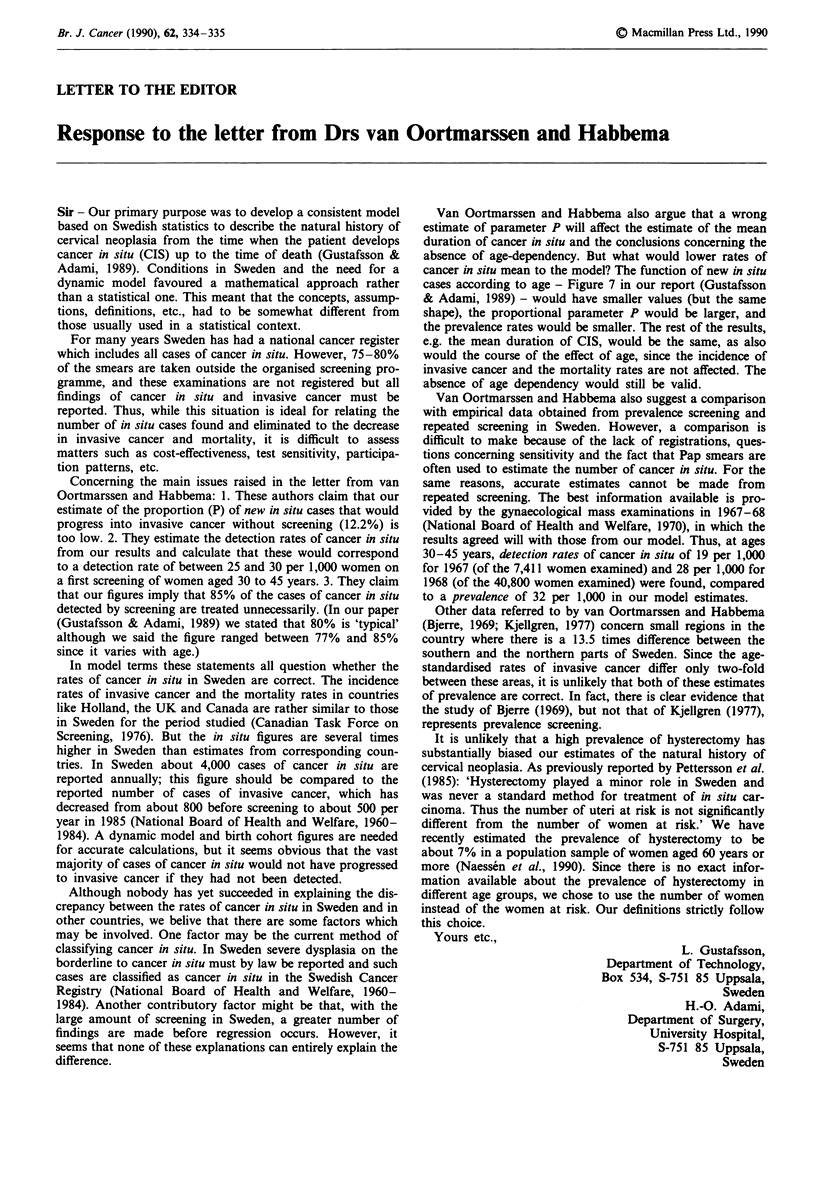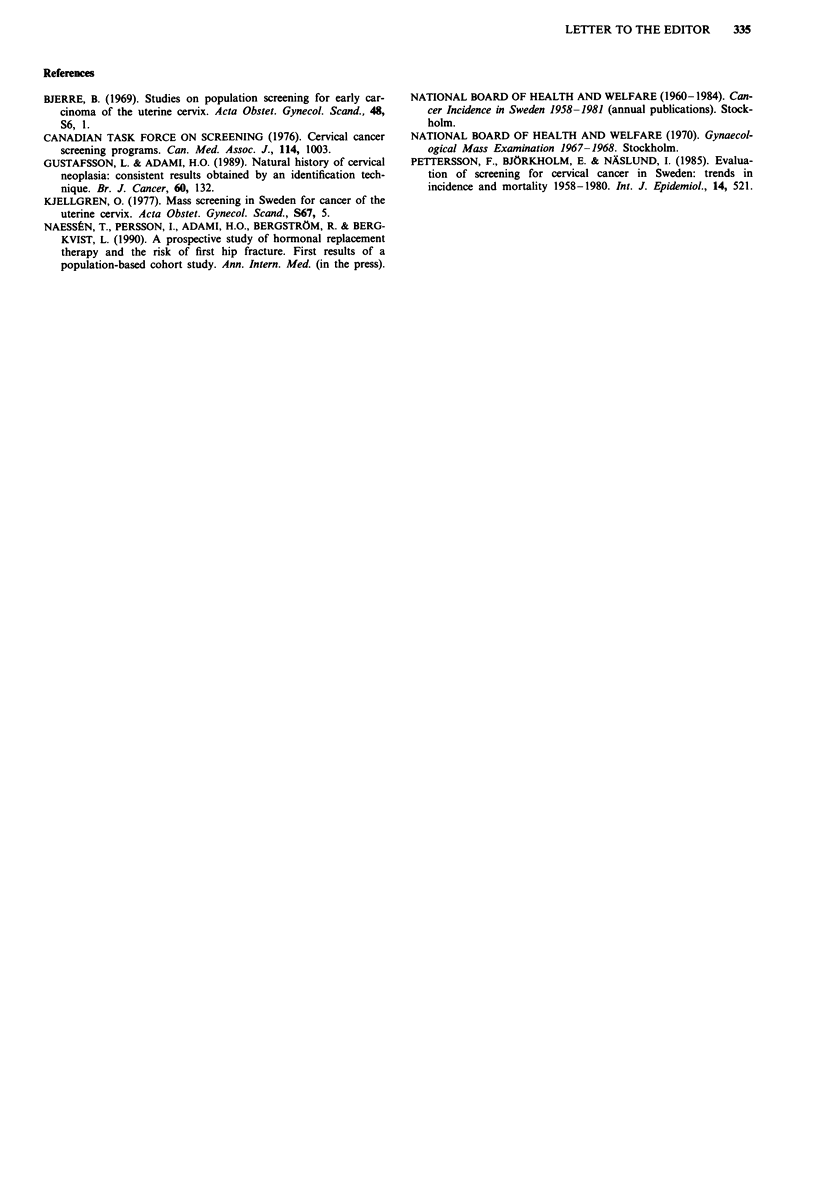# Response to the letter from Drs Oortmarssen and Habbema

**Published:** 1990-08

**Authors:** L. Gustafsson, H.-O. Adami


Br. J. Cancer (1990), 62, 334–335 © Macmillan Press Ltd., 1990

LETTER TO THE EDITOR

### Response to the letter from Drs van Oortmarssen and Habbema

**Sir** – Our primary purpose was to develop a consistent model based on Swedish statistics to describe the natural history of cervical neoplasia from the time when the patient develops cancer *in situ* (CIS) up to the time of death (Gustafsson & Adami, 1989). Conditions in Sweden and the need for a dynamic model favoured a mathematical approach rather than a statistical one. This meant that the concepts, assumptions, definitions, etc., had to be somewhat different from those usually used in a statistical context.

For many years Sweden has had a national cancer register which includes all cases of cancer *in situ*. However, 75–80% of the smears are taken outside the organised screening programme, and these examinations are not registered but all findings of cancer *in situ* and invasive cancer must be reported. Thus, while this situation is ideal for relating the number of *in situ* cases found and eliminated to the decrease in invasive cancer and mortality, it is difficult to assess matters such as cost-effectiveness, test sensitivity, participation patterns, etc.

Concerning the main issues raised in the letter from van Oortmarssen and Habbema: 1. These authors claim that our estimate of the proportion (P) of *new in situ* cases that would progress into invasive cancer without screening (12.2%) is too low. 2. They estimate the detection rates of cancer *in situ* from our results and calculate that these would correspond to a detection rate of between 25 and 30 per 1,000 women on a first screening of women aged 30 to 45 years. 3. They claim that our figures imply that 85% of the cases of cancer *in situ* detected by screening are treated unnecessarily. (In our paper (Gustafsson & Adami, 1989) we stated that 80% is 'typical' although we said the figure ranged between 77% and 85% since it varies with age.)

In model terms these statements all question whether the rates of cancer *in situ* in Sweden are correct. The incidence rates of invasive cancer and the mortality rates in countries like Holland, the UK and Canada are rather similar to those in Sweden for the period studied (Canadian Task Force on Screening, 1976). But the *in situ* figures are several times higher in Sweden than estimates from corresponding countries. In Sweden about 4,000 cases of cancer *in situ* are reported annually; this figure should be compared to the reported number of cases of invasive cancer, which has decreased from about 800 before screening to about 500 per year in 1985 (National Board of Health and Welfare, 1960–1984). A dynamic model and birth cohort figures are needed for accurate calculations, but it seems obvious that the vast majority of cases of cancer *in situ* would not have progressed to invasive cancer if they had not been detected.

Although nobody has yet succeeded in explaining the discrepancy between the rates of cancer *in situ* in Sweden and in other countries, we belive that there are some factors which may be involved. One factor may be the current method of classifying cancer *in situ*. In Sweden severe dysplasia on the borderline to cancer *in situ* must by law be reported and such cases are classified as cancer *in situ* in the Swedish Cancer Registry (National Board of Health and Welfare, 1960–1984). Another contributory factor might be that, with the large amount of screening in Sweden, a greater number of findings are made before regression occurs. However, it seems that none of these explanations can entirely explain the difference.

Van Oortmarssen and Habbema also argue that a wrong estimate of parameter $P$ will affect the estimate of the mean duration of cancer *in situ* and the conclusions concerning the absence of age-dependency. But what would lower rates of cancer *in situ* mean to the model? The function of new *in situ* cases according to age – Figure 7 in our report (Gustafsson & Adami, 1989) – would have smaller values (but the same shape), the proportional parameter $P$ would be larger, and the prevalence rates would be smaller. The rest of the results, e.g. the mean duration of CIS, would be the same, as also would the course of the effect of age, since the incidence of invasive cancer and the mortality rates are not affected. The absence of age dependency would still be valid.

Van Oortmarssen and Habbema also suggest a comparison with empirical data obtained from prevalence screening and repeated screening in Sweden. However, a comparison is difficult to make because of the lack of registrations, questions concerning sensitivity and the fact that Pap smears are often used to estimate the number of cancer *in situ*. For the same reasons, accurate estimates cannot be made from repeated screening. The best information available is provided by the gynaecological mass examinations in 1967–68 (National Board of Health and Welfare, 1970), in which the results agreed will with those from our model. Thus, at ages 30–45 years, *detection rates* of cancer *in situ* of 19 per 1,000 for 1967 (of the 7,411 women examined) and 28 per 1,000 for 1968 (of the 40,800 women examined) were found, compared to a *prevalence* of 32 per 1,000 in our model estimates.

Other data referred to by van Oortmarssen and Habbema (Bjerre, 1969; Kjellgren, 1977) concern small regions in the country where there is a 13.5 times difference between the southern and the northern parts of Sweden. Since the age-standardised rates of invasive cancer differ only two-fold between these areas, it is unlikely that both of these estimates of prevalence are correct. In fact, there is clear evidence that the study of Bjerre (1969), but not that of Kjellgren (1977), represents prevalence screening.

It is unlikely that a high prevalence of hysterectomy has substantially biased our estimates of the natural history of cervical neoplasia. As previously reported by Pettersson *et al.* (1985): 'Hysterectomy played a minor role in Sweden and was never a standard method for treatment of *in situ* carcinoma. Thus the number of uteri at risk is not significantly different from the number of women at risk.' We have recently estimated the prevalence of hysterectomy to be about 7% in a population sample of women aged 60 years or more (Naessén *et al.*, 1990). Since there is no exact information available about the prevalence of hysterectomy in different age groups, we chose to use the number of women instead of the women at risk. Our definitions strictly follow this choice.

Yours etc.,

L. Gustafsson,
Department of Technology,
Box 534, S-751 85 Uppsala,
Sweden
H.-O. Adami,
Department of Surgery,
University Hospital,
S-751 85 Uppsala,
Sweden

### References

BJERRE, B. (1969). Studies on population screening for early carcinoma of the uterine cervix. *Acta Obstet. Gynecol. Scand.*, **48**, S6, 1.

CANADIAN TASK FORCE ON SCREENING (1976). Cervical cancer screening programs. *Can. Med. Assoc. J.*, **114**, 1003.

GUSTAFSSON, L. & ADAMI, H.O. (1989). Natural history of cervical neoplasia: consistent results obtained by an identification technique. *Br. J. Cancer*, **60**, 132.

KJELLGREN, O. (1977). Mass screening in Sweden for cancer of the uterine cervix. *Acta Obstet. Gynecol. Scand.*, **S67**, 5.

NAESSÉN, T., PERSSON, I., ADAMI, H.O., BERGSTRÖM, R. & BERGKVIST, L. (1990). A prospective study of hormonal replacement therapy and the risk of first hip fracture. First results of a population-based cohort study. *Ann. Intern. Med.* (in the press).

NATIONAL BOARD OF HEALTH AND WELFARE (1960–1984). *Cancer Incidence in Sweden 1958–1981* (annual publications). Stockholm.

NATIONAL BOARD OF HEALTH AND WELFARE (1970). *Gynaecological Mass Examination 1967–1968*. Stockholm.

PETTERSSON, F., BJÖRKHOLM, E. & NÄSLUND, I. (1985). Evaluation of screening for cervical cancer in Sweden: trends in incidence and mortality 1958–1980. *Int. J. Epidemiol.*, **14**, 521.